# Menin Inhibition in Acute Myeloid Leukemia: Rewiring Leukemic Transcriptional Networks

**DOI:** 10.3390/ijms27114886

**Published:** 2026-05-28

**Authors:** Ali Tarhini, Michael Romanos, Aref Al-Kali, Antoine N. Saliba

**Affiliations:** 1 Department of Oncology, Mayo Clinic, Rochester, MN 55905, USA; tarhini.ali@mayo.edu; 2 Department of Medicine, Mayo Clinic, Rochester, MN 55905, USA; romanos.michael@mayo.edu; 3 Division of Hematology, Department of Medicine, Mayo Clinic, Rochester, MN 55905, USA

**Keywords:** menin inhibitors, acute myeloid leukemia, *HOXA*/*MEIS1*, *KMT2A* rearrangement, *NPM1* mutation

## Abstract

Among the transcriptional dependencies that sustain leukemic identity in acute myeloid leukemia (AML), the menin–KMT2A chromatin complex has emerged as a central regulatory node. The scaffold protein menin, encoded by *MEN1*, facilitates transcriptional activation of HOX and MEIS family genes during normal hematopoietic development. In AML, this physiologic and developmentally regulated role is co-opted to sustain constitutive HOX/MEIS-driven programs that block differentiation and maintain leukemic potential. Although dependency on menin is most clearly established in *KMT2A*-rearranged and *NPM1*-mutated AML, this vulnerability appears to arise from a shared transcriptional state characterized by persistent HOX activation rather than from any single genetic alteration. Pharmacologic disruption of the menin-KMT2A interaction collapses stemness-associated transcriptional networks, promotes myeloid differentiation, and attenuates leukemic self-renewal. Clinical activity observed with menin inhibitors provides translational validation of this dependency and establishes menin inhibition as a differentiation-based therapeutic strategy. In this review, we examine the molecular basis of menin-dependent transcriptional regulation in AML and its implications for therapeutic targeting with menin inhibitors and resistance to therapy.

## 1. Introduction

Nucleophosmin 1 (*NPM1*) mutated acute myeloid leukemia (AML) is among the most common AML subtypes, present in 30–35% of newly diagnosed cases [1,2]. While less common and occurring in 5–15% of adult AML cases, Lysine Methyltransferase 2A (KMT2A)-rearranged (*KMT2A*r) AML is associated with dismal outcomes especially in the relapsed/refractory (R/R) setting [3,4,5,6]. Both AML subtypes converge on the aberrant activation of transcriptional programs involving the *HOXA* (homeobox A) cluster genes and *MEIS1* (myeloid ecotropic viral integration site 1) [7,8,9,10]. Menin, a scaffold protein encoded by *MEN1*, operates within the *KMT2A*, formerly known as *MLL1*, chromatin complex. The interaction of menin with the N-terminus of KMT2A is central to *HOXA* and *MEIS1* expression and subsequent leukemogenesis [11,12]. While AML classification has been largely defined and treated according to specific mutations and molecular alterations, accumulating evidence has shown that the leukemic stem cell epigenetic signature can be mutation-independent, with several genetically distinct mutations converging on a shared *HOXA* and *MEIS1* transcription cluster [8,13,14], with menin at the center of this activation. In this context, genetic lesions function less as isolated determinants of leukemic behavior and more as gateways into a shared transcription circuit that defines and maintains AML identity.

A long history of scientific discoveries in leukemia laid the foundation for the clinical development of menin inhibitors. Almost 15 years after the discovery of a t(4;11) translocation in acute lymphoblastic leukemia (ALL) in 1977 [15,16], several other translocations involving chromosome 11q23 were identified in both ALL and AML. These rearrangements produce breakpoint junctions within 11q23 that disrupt a gene spanning this region. This gene was named *MLL* [17,18,19], known today as *KMT2A*. Subsequent functional studies provided insight into the biological role of this gene. In mouse models lacking *Mll*, expression of *Hoxa-7* and *Hoxa-9* was essentially abrogated, demonstrating that *KMT2A* is required for activation of *HOXA* gene expression [20,21]. These findings led to the hypothesis that aberrant KMT2A fusion proteins could drive oncogenesis and leukemic transformation [20,21]. In the early 2000s, gene expression profiling studies further established that leukemias harboring *KMT2A* rearrangement exhibit a distinct transcriptional signature characterized by aberrant activation of *HOXA* cluster genes and the *MEIS1* cofactor [22,23,24]. A few years later, menin was identified as a core component of the KMT2A complex, with its deletion causing loss of KMT2A activity [25,26]. Shortly thereafter, additional work revealed that the menin–KMT2A interaction is required for KMT2A-driven leukemogenesis [27]. In the early 2010s, structural studies identified the binding interface between menin and the KMT2A protein [28,29,30], opening the door to the development of small-molecule inhibitors targeting the menin–KMT2A interaction [31]. The therapeutic relevance of this strategy became increasingly apparent when subsequent epigenomic studies revealed that the *HOXA* transcription program is not restricted to *KMT2A*r AML but is shared across several AML subtypes, including the relatively common subtype of *NPM1*-mutated (*NPM1*m) AML [32,33,34]. These discoveries ultimately paved the way for the development of small-molecule menin inhibitors, a detailed discussion of these therapeutic advances will be revisited in Section 5. Before addressing the translational implications of targeting menin, we will first consider the biological foundations that established menin as a central regulator of leukemogenic transcriptional programs.

Accordingly, in this review, we examine the molecular basis of menin interactions with genes such as *KMT2A* and *NPM1*, how these interactions upregulate the *HOXA* and *MEIS1* downstream pathways, and how these mechanisms explain the therapeutic benefits of targeted menin inhibition and the emergence of resistance.

## 2. Menin in Normal Hematopoiesis

### 2.1. MEN1 Protein Structure and Function

Menin is known to be a tumor suppressor scaffold protein that is linked to multiple endocrine neoplasia 1 (MEN1) syndrome. In humans, *MEN1*, a gene located on chromosome 11q13, comprises nine introns and ten exons and produces multiple transcript variants with 5′-UTR heterogeneity, though these encode the same menin protein. Menin is a predominantly nuclear *α*-helical, 610-amino acid protein with its core containing three tetratricopeptide repeat (TPR) motifs flanked by additional helical bundles [35]. The crystal structure of menin reveals a curved, hand-like conformation composed of distinct structural domains. The N-terminal region forms an extended *β*-hairpin and contributes to a transglutaminase-like domain resembling a “thumb,” while the central portion adopts a “palm” configuration that contains a deep binding pocket accommodating KMT2A-derived peptides, and the C-terminal region resembles “finger”-like projections that harbor nuclear localization signals [28,29,36]. This structural organization enables menin to function as a scaffold protein, mediating interactions not only with KMT2A but also with additional partners involved in transcriptional regulation and tumor suppression, including JunD, SMAD3, and MYC [35,36,37,38,39]. Importantly, although menin classically functions as a tumor suppressor in endocrine tissues, it serves as a critical oncogenic cofactor in leukemogenesis.

### 2.2. Menin-KMT2A Chromatin Complex

As mentioned above, menin interacts with many proteins, including KMT2A, a large protein central to normal development and hematopoiesis [40,41]. Menin influences KMT2A interaction with DNA, and the menin-KMT2A chromatin complex is essential for the sustained transcriptional activation of the HOXA cluster genes and *MEIS1* [11,25,27,42].

Menin functions as an adaptor protein that bridges the H3K4 histone methyltransferase KMT2A to chromatin. The menin-KMT2A complex methylates H3K4 at the *HOXA9* locus, leading to the transcription of *HOXA* genes [43,44,45]. In addition to its interaction with KMT2A, menin associates with other epigenetic regulators, including protein arginine methyltransferase 5 (PRMT5) and the H3K9 methyltransferase (SUV39H1), as well as multiple transcription factors, thereby participating in diverse chromatin-modifying and transcriptional complexes [43,44,45]. HOXA proteins regulate normal hematopoiesis through coordinated transcriptional programs that maintain stem and progenitor cell integrity, control lineage commitment, and establish the epigenetic framework of blood development [46,47]. HOXA9 plays a central role in sustaining hematopoietic stem cells (HSC) by promoting proliferation and self-renewal, further amplified by cooperative positive feedback loops involving other *HOXA* genes that reinforce overall HOXA cluster expression [48,49,50,51], and *HOXA9* is tightly regulated with *MEIS1* [52].

Hence, the menin-KMT2A-HOXA axis plays a critical role in early hematopoietic development and stem cell maintenance. Its role in mature hematopoiesis appears less critical, whereas a greater dependence is more characteristic of leukemic states [27]. Dysregulated activation of this pathway can lead to leukemogenic transformation and AML development [8,22,53].

## 3. HOXA/MEIS1 in AML: Menin Dependency

### 3.1. HOXA9/MEIS1 Axis in Leukemic Self-Renewal

*HOXA9* and *MEIS1* are key regulators of HSC self-renewal and are controlled by upstream regulators, including the menin–KMT2A interaction, which is particularly important in leukemic contexts. Aberrant activation of the menin–KMT2A complex disrupts the temporal control over HOXA gene networks and supports leukemogenesis [54]. The progression and maintenance of these leukemic profiles is dependent on *MEIS1* co-activity with *HOXA9* [55,56]. In experimental models, dysregulated *HOXA9* expression promotes leukemic self-renewal in part through repression of the *Ink4a*/*b* locus and activation of pro-survival pathways, including BCL2- and IGF1-related programs [49,51,57,58]. *MEIS1* is vital to *HOXA9* function; it localizes to *HOXA9*-preactivated enhancers and maintains the leukemic transcription program by suppressing granulocytic differentiation, thereby supporting indefinite self-renewal [59,60]. With this intricate network of genetic interaction, aberrant *HOXA9* expression, at the core of this network, is associated with clinically aggressive disease features and poor patient outcomes in AML, including decreased survival, higher risk of relapse, and poor treatment response [61,62,63,64].

### 3.2. Genotype as Proxy for Transcriptional Dependency

**Figure 1:**
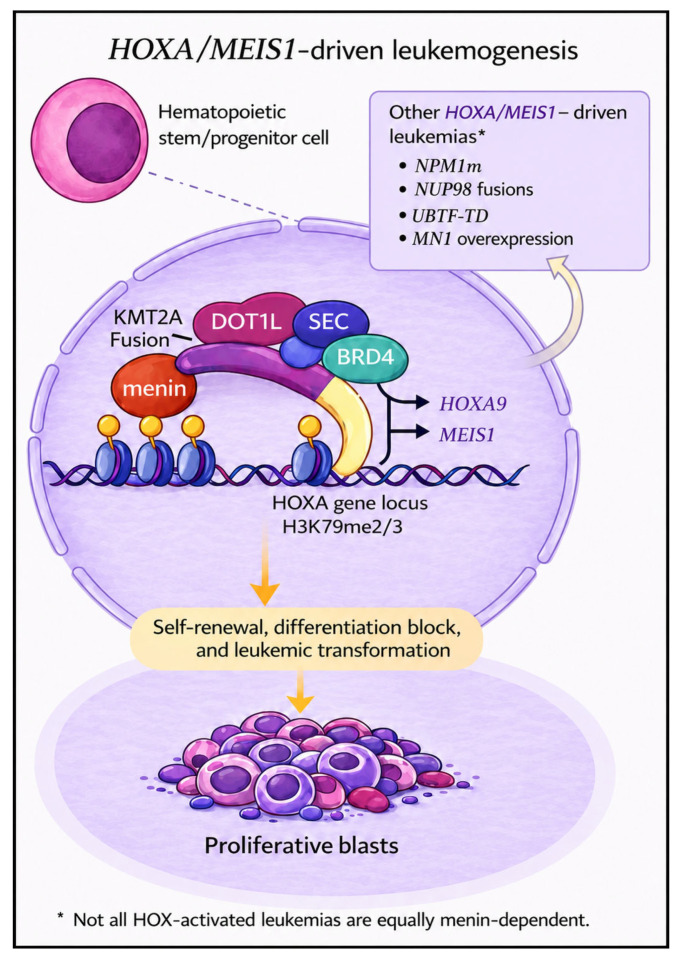
*HOXA9*/*MEIS1* driven leukemogenesis in acute myeloid leukemia.

*HOXA9* serves as a central transcription node in AML (Figure 1), where multiple upstream distinct genetic alterations ultimately converge on its activation. The genetic alterations driving AML through HOXA dysregulation can be either KMT2A-dependent or KMT2A-independent [8]. However, it is worth noting that not all AML subtypes exhibit HOXA9 overexpression [63]. *KMT2A* rearrangement (*KMT2A*r) is a recurrent chromosomal abnormality in AML, characterized by fusion of the *KMT2A* gene with one of more than 100 different partners identified, with each partner distinctly influencing the leukemia phenotype and severity [65,66]. In this section, we delineate how distinct genetic alterations in AML converge upon a shared transcriptional dependency centered on *HOXA*/*MEIS1* activation, and we summarize the mechanistic role of menin within this regulatory axis.

#### 3.2.1. KMT2A-Rearranged AML

*KMT2A* rearrangements generate fusion oncoproteins that enforce persistent transcription of *HOXA9* and *MEIS1* [67]. These fusions (e.g., *KMT2A*::*AF9*, *KMT2A*::*ENL*, *KMT2A*::*AF10*) retain the N-terminal *KMT2A* domain that mediates interaction with menin, which is required for chromatin recruitment [68]. The fusion complexes occupy broad regions across the HOXA locus and promote transcription through coordinated epigenetic remodeling, including H3K4 methylation and DOT1L-mediated H3K79 methylation. Disruption of the menin–KMT2A interaction results in rapid downregulation of *HOXA*/*MEIS1* expression and promotes leukemic differentiation, underscoring the strong dependence of this subtype on the menin-KMT2A axis [69,70,71,72,73].

#### 3.2.2. *NPM1*-Mutated AML

*NPM1* mutations are recurrent frameshift alterations predominantly involving exon 12 that produce two critical C-terminal changes: (i) loss of the nucleolar localization signal and (ii) acquisition of a novel nuclear export signal (NES). Wild-type NPM1 is only weakly exported by the export protein Chromosomal Maintenance 1 (CRM1) under physiologic conditions, whereas mutant NPM1 (NPM1c) forms efficient complexes with CRM1 as a consequence of the acquired NES. The most common *NPM1* mutations disrupt tryptophan residues at positions 288 and 290 (or W290 alone), residues required for nucleolar retention [74,75]. Mutants retaining W288 consistently pair with stronger NES motifs, suggesting selective pressure favoring maximal cytoplasmic accumulation [75]. The frameshift generates a leucine-rich C-terminal NES [76], enabling efficient recognition by the nuclear export receptor XPO1/CRM1 and active cytoplasmic transport [77].

Despite its predominant cytoplasmic localization, a residual nucleoplasmic fraction of NPM1c retains direct chromatin binding at leukemogenic loci, including active promoters within the *HOXA*/*B* clusters and *MEIS1*, where it co-occupies chromatin with *KMT2A* [9,34,78,79,80]. NPM1c sustains transcriptional activation in part through inhibition of histone deacetylase (HDAC) activity and maintenance of activating histone modifications [9,33,81]. These interactions contribute to the formation of a transcriptional hub that recruits RNA polymerase II to target genes. Targeted degradation of NPM1c results in rapid loss of RNA polymerase II occupancy and activating histone marks at these loci [9,78]. The leukemic state is highly dependent on cytoplasmic NPM1c. Loss of NPM1c from the cytoplasm—either through nuclear relocalization via XPO1 inhibition (e.g., selinexor) or through targeted degradation—leads to rapid downregulation of HOX gene expression, induction of myeloid differentiation, and prolonged survival in murine leukemia models [7].

Additionally, NPM1c reshapes CTCF-dependent topologically associated domains (TADs), altering three-dimensional chromatin architecture to promote activation of HOX genes and reprogram cell cycle regulatory networks [82].

#### 3.2.3. *NUP98* Fusions

*NUP98* fusions, most commonly *NUP98*::*HOXA9* and *NUP98*::*NSD1*, are recurrent gene fusions in AML. These fusions function as aberrant transcription factors in AML by combining the intrinsically disordered Phe-Gly (FG)-repeat domain of NUP98 with partner proteins involved in transcriptional regulation. The FG repeats act as potent transactivation domains that recruit CBP/p300 and other coactivators, driving transcriptional activation of HOX-responsive genes [83]. In the case of *NUP98*::*HOXA9*, chromatin-prebound CRM1 (*XPO1*) contributes to selective recruitment of the fusion protein to HOXA cluster regions, leading to sustained *HOXA9* and *MEIS1* activation [84,85,86]. In the case of *NUP98*::*NSD1*, fusion-mediated recruitment of NSD1 enforces H3K36 methylation and an active chromatin state while preventing EZH2-driven repression at the *HOXA* locus, thereby sustaining aberrant *HOXA7*/*HOXA9*/*MEIS1* expression during differentiation [87,88]. NUP98 fusion proteins engage epigenetic machinery to stabilize the leukemic program. They physically require wild-type KMT2A and menin to localize to HOX promoters and maintain leukemogenesis, and menin inactivation reverses the transcriptional program driven by *NUP98* fusions [89,90,91]. Therefore, due to the role of menin in the *KMT2A*–*HOXA9* axis, menin inhibition is being explored as an emerging treatment option for AML patients with *NUP98* fusions [92,93,94].

#### 3.2.4. Other Genetic Alterations

Beyond these major alterations, multiple less common genetic variations utilize the *HOXA9*/*MEIS1* pathway, either in a KMT2A-dependent or independent manner. KMT2A-dependent alterations include *NUP214* rearrangements [95], *MN1* overexpression [96,97,98], *UBTF* tandem duplications [99,100], *CALM*::*AF10* fusions [101,102], and *MOZ* fusions [103,104]. Despite their diverse upstream mechanisms, these KMT2A-dependent alterations, whether through direct activation or through loss of repression, converge on the common outcome of sustained *HOXA9*/*MEIS1* expression. HOXA expression signature itself resembles that of hematopoietic stem/progenitor cells (HSPCs), but it is aberrantly maintained during differentiation [32].

## 4. Molecular Mechanisms of Menin Inhibition

**Figure 2:**
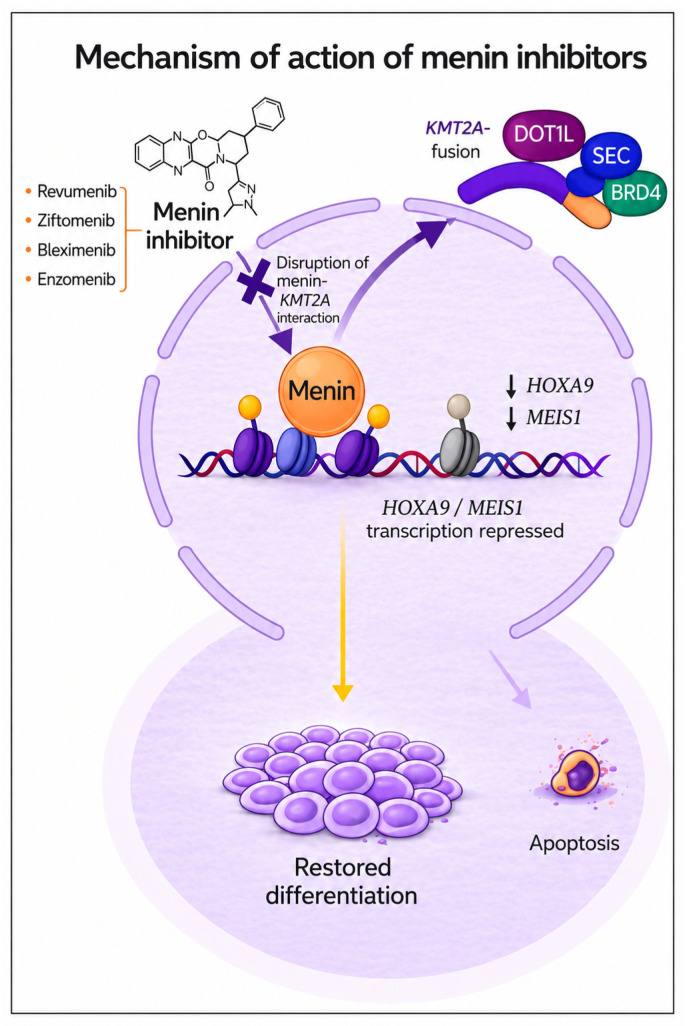
Mechanism of action of menin inhibitors.

Considering the critical role that menin plays in sustaining KMT2A/menin-dependent leukemogenesis, therapeutic inhibition of the menin–KMT2A interaction has emerged as a promising treatment strategy for AML and ALL (Figure 2). Menin inhibition disrupts the menin–KMT2A interaction, thereby disassembling KMT2A complexes from the *HOXA9* loci. This leads to the collapse of *HOXA9*/*MEIS1*-driven transcription and downregulation of other menin–KMT2A dependent factors [31]. Hence, menin inhibition promotes cell-cycle arrest, induces myeloid differentiation, reduces clonogenic potential, and, in some contexts, enhances apoptosis [31].

Beyond HOXA downregulation, menin inhibition can also disrupt the maintenance of leukemic identity. For example, the menin–KMT2A fusion interaction has been found to prevent the binding of a KMT2C/KMT2D–UTX chromatin regulatory complex to target gene promoters, thus blocking activation of differentiation-associated and tumor suppressive programs [105]. Menin inhibition releases this block, allowing KMT2C/KMT2D–UTX to activate these tumor suppressive genes. Moreover, menin blockade also induces expression of human endogenous retroviral elements and upregulation of interferon-stimulated genes, which enhance major histocompatibility complex II (MHC-II) presentation in *KMT2A*r and *NPM1*m AML cells and facilitate T-cell mediated clearance [106]. Collectively, these effects lead to collapse of leukemic stem cell transcriptional programs and shift transcriptional reprogramming toward myeloid differentiation, forming the basis for the clinical development of selective menin inhibitors for several genetically defined AML subtypes, including *KMT2A*r, *NPM1*m, and *NUP98*r AML, among others.

## 5. Clinical Translation as Proof of Mechanism

From there on, compelling evidence of the antileukemic efficacy of these menin inhibitors began to emerge. Landmark preclinical studies published in 2020 showed that the small-molecule menin inhibitors VTP-50469 and MI-3454 induced differentiation, promoted apoptosis in susceptible models, and led to leukemia regression or remission in patient-derived xenograft models of *KMT2A*r and *NPM1*m AML, with relative sparing of normal hematopoiesis [69,107,108]. Clinical development proceeded in parallel through the AUGMENT-101 study of revumenib (SNDX-5613) and the KOMET-001 study of ziftomenib (KO-539). Early and subsequent trial data demonstrated clinically meaningful activity in heavily pretreated R/R *KMT2A*r and *NPM1*m AML, including complete remissions (CR) and measurable residual disease (MRD) negativity in responding patients [109,110,111]. These advances led to accelerated U.S. FDA approval of revumenib for R/R acute leukemia with a *KMT2A*r in November 2024 [112], followed in 2025 by expansion of the revumenib approval and the first approval for ziftomenib in R/R AML with susceptible *NPM1* mutations [113,114]. Building on these regulatory approvals, ongoing studies are evaluating additional menin inhibitors, earlier-line use, combination regimens with various drugs including venetoclax and hypomethylating agents, and activity in other *HOXA*/*MEIS1*-dependent leukemic genotypes. In this section, we discuss menin-inhibitors, both approved and in development, and review their mechanisms of action, clinical activity, contraindications, and safety profiles (Table 1).

### 5.1. Revumenib

Revumenib (SNDX-5613) is an oral small-molecule menin inhibitor evaluated in the pivotal AUGMENT-101 phase 1/2 study (NCT04065399) in heavily pretreated R/R acute leukemias (AML or ALL) harboring *KMT2A*r or *NPM1*m [110,115,117]. Revumenib monotherapy demonstrated clinical activity across cohorts. The clinical efficacy results and adverse event profile are summarized in Table 1.

In 97 efficacy-evaluable pediatric and adult patients with R/R *KMT2A*r acute leukemia treated at the recommended phase 2 dose (RP2D), revumenib achieved a composite CR (CR + CRh) rate of 23% and an overall response rate (ORR) of 63%, with MRD negativity by flow cytometry in 61% of responders [115]. The median time to response was 1.9 months, and the median duration of response (DoR) was 6.4 months. Approximately 40% of responding patients subsequently underwent allogeneic hematopoietic stem cell transplantation (alloSCT), while those who did not proceed to alloSCT had a median overall survival (OS) of 8.0 months.

In a separate *NPM1*m cohort (n = 64) treated at the same dose, revumenib produced a CR + CRh rate of 23% and an ORR of 47%, with MRD negativity in 64% of responders and a median DoR of 4.7 months [117]. Similarly, 40% of responders proceeded to alloSCT. Among patients previously exposed to venetoclax, revumenib achieved a CR + CRh rate of 17% and an ORR of 41% [117].

From a safety standpoint, revumenib has a manageable toxicity profile, although attention to drug–drug interactions and QTc monitoring is required. Revumenib is a CYP3A4 substrate, and dose adjustments are recommended with concomitant strong CYP3A4 inhibitors (such as azole antifungals) [110,122]. The emergence of differentiation syndrome (DS) in early trials was interpreted as clinical evidence that menin inhibition can reverse the differentiation block central to *KMT2A*r and *NPM1*m leukemias [65], and QT prolongation was identified as a dose-limiting toxicity [110]. Based on the FDA label for revumenib (n = 241 patients), DS occurred in 25% of patients (all grades), with grade 3/4 in 12% and fatal DS in 2 patients (<1%) [122]. QTc prolongation occurred in 36% of patients, with grade 3 in 15% and grade 4 in 2% [122].

Other commonly reported adverse events included nausea (48%), bleeding (48%), neutropenic fever (37%), diarrhea (29%), and infection (29%) [122]. Serious adverse events occurred in 76% of patients, including infection, neutropenic fever, DS, and serious hemorrhage [122]. Despite these toxicities, most events were manageable with supportive care, monitoring, and dose modification. Dose interruption was required in close to 50% of patients, most commonly for QTc prolongation, infection, neutropenic fever, and DS [122]. Despite class-related toxicities such as DS and QTc prolongation, the safety profile of revumenib is manageable relative to its clinical activity, supporting its U.S. FDA approval in November 2024 for R/R *KMT2A*r acute leukemia and in October 2025 for R/R *NPM1*m AML.

### 5.2. Ziftomenib

The activity of ziftomenib (KO-539), another oral small-molecule menin inhibitor, was evaluated in the phase 1b/2 KOMET-001 trial (NCT04067336) in patients with R/R AML [118]. The RP2D of 600 mg once daily does not require modification in the setting of concomitant azole antifungals and, unlike revumenib, appears to have a more favorable drug–drug interaction profile [118]. However, QT prolongation remains a labeled risk.

In the pivotal phase 2 expansion cohort of 92 heavily pretreated adults with NPM1m AML, single-agent ziftomenib produced a composite CR rate of 22% and an ORR of 33%. Median DoR was 4.6 months, with a median OS of 6.6 months across the cohort [123]. Patients who achieved remission had substantially improved outcomes, with a median OS of 16.4 months. Responses were observed regardless of *FLT3* or *IDH* co-mutations and were maintained in patients previously treated with venetoclax-based regimens. Among individuals achieving CR or CRh (CR with partial hematologic recovery), approximately 61% achieved MRD negativity. These results paved the way for U.S. FDA approval of ziftomenib for R/R *NPM1*m AML [124].

A smaller phase 1 cohort of *KMT2A*r AML (n = 20) demonstrated an ORR of 20% and CR/CRh rate of 10%, with a similar DoR [118]. The rate and severity of DS led to halting enrollment for *KMT2A*r AML. Since then, development has increasingly focused on combination strategies, including ongoing studies evaluating ziftomenib with standard therapies such as venetoclax and hypomethylating agents, intensive 7 + 3 chemotherapy, and gilteritinib (KOMET-007, NCT05735184).

### 5.3. Bleximenib

Bleximenib/JNJ-75276617, an oral, selective menin inhibitor, downregulates the expression of key leukemogenic genes including *MEIS1* and *FLT3* [125]. Beyond direct antileukemic effects, bleximenib promotes epigenetic activation of CIITA, resulting in increased MHC class I and II expression and enhanced T-cell–mediated killing of leukemic blasts [126]. Bleximenib binds menin at a site distinct from revumenib and retains activity in models harboring *MEN1* resistance mutations, such as M327I and T349M [125].

Clinically, bleximenib monotherapy has been evaluated in a phase 1 cAMeLot-1 trial (NCT04811560) in R/R *KMT2A*r and *NPM1*m acute leukemias. Preliminary data have shown a CR/CRh rate and ORR in the range of 35% and 50% respectively [120]. Adverse events include DS, neutropenia, and thrombocytopenia, while only one grade 3 QTc prolongation event was observed [120].

### 5.4. Enzomenib

Enzomenib (DSP-5336) is another oral menin inhibitor being studied in the ongoing phase 1/2 study (NCT04988555). Among patients with *KMT2A*r leukemia (n = 22), the ORR was 59%, with CR/CRh achieved in 23%. In those with *NPM1*m AML (n = 13), the ORR was 54%, including CR/CRh in 23%. Responses occurred rapidly with a median of 1 month. Enzomenib therapy was associated with a relatively low incidence of DS (11%), and azole antifungal coadministration did not significantly impact drug exposure [121].

### 5.5. Other Emerging Agents

Several other menin inhibitors are in early-stage preclinical and clinical development. Efforts focus on improving durability, mitigating QTc prolongation, limiting CYP3A4 interactions, and overcoming resistance mutations. These agents include BMF-219, a covalent menin inhibitor (COVALENT-101, NCT05153330) [127] and BN104, a non-covalent menin inhibitor with encouraging early activity and manageable toxicity (NCT06052813) [128]. Although clinical data for these newer menin inhibitors remain limited, preclinical studies suggest that several compounds may improve compared to earlier agents by retaining activity against acquired *MEN1* resistance mutations and by reducing QTc prolongation [129,130,131,132,133]. Whether these advantages translate into clinically meaningful benefit will require further validation in human studies.

### 5.6. Clinical Considerations When Interpreting Menin Inhibitor Trial Data

Given that menin inhibitors first entered human clinical investigation less than a decade ago and the first FDA approvals have only occurred within the past two years, the bulk of the available data is derived from early-phase, single-arm, and non-randomized clinical trials. These trials are inherently susceptible to selection bias, small sample sizes, and heterogeneity in patient populations. Cross-trial comparisons between different menin inhibitors should therefore be approached with caution, where differences in efficacy or toxicity may reflect differences in trial design and eligibility rather than true pharmacologic distinctions among agents. Randomized comparative studies and longer-term follow-up will be required to definitively establish the relative efficacy and safety profiles of menin inhibitors in AML.

Moreover, despite the emerging data generated from clinical trials in progress, identifying a single preferred menin inhibitor for all settings and in all patients will likely prove near-impossible. We envision a new reality in HOXA-driven AML where there is an embarrassment of riches just like in chronic myeloid leukemia and ABL-tyrosine kinase inhibitors. Those decisions will need to account for patient comorbidities, preferred dosing schedules, concomitant medications (especially acid suppressants and azole antifungals), AML subtype, goals of therapy (palliative versus curative), and, down the line, the combination backbone. While current data suggest comparable efficacy across the drug class, the meaningful distinctions to date relate to toxicity and drug–drug interactions, and these are likely to change as we learn more about these agents.

## 6. Toxicity

**Figure 3:**
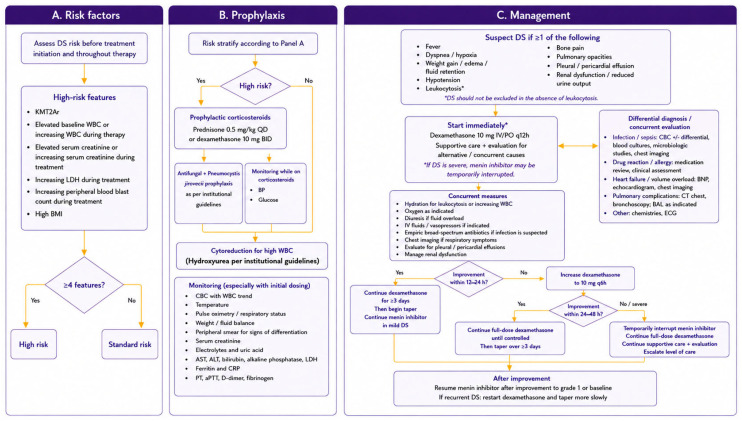
Proposed algorithm for identification, prevention, and management of menin inhibitor- associated differentiation syndrome. aPTT, activated partial thromboplastin time; ALT, alanine aminotransferase; AST, aspartate aminotransferase; BAL, bronchoalveolar lavage; BID, twice daily; BMI, body mass index; BNP, B-type natriuretic peptide; BP, blood pressure; CBC, complete blood count; CRP, C-reactive protein; CT, computed tomography; DS, differentiation syndrome; ECG, electrocardiogram; IV, intravenous; *KMT2A*r, *KMT2A* rearrangement; LDH, lactate dehydrogenase; PO, by mouth/orally; PT, prothrombin time; QD, once daily; q6h, every 6 h; q12h, every 12 h; WBC, white blood cell count. * Special clinical considerations within the proposed algorithm.

As discussed with the individual agents, menin inhibitors demonstrate an overall manageable safety profile, with several toxicities reflecting either on-target differentiation biology or molecule-specific pharmacologic effects. DS, a class-defining toxicity resulting from rapid leukemic blast maturation, occurs in approximately 10–20% of patients receiving menin inhibitor monotherapy and is generally managed with corticosteroids and temporary treatment interruption when recognized early (Figure 3) [117,118]. Although combination regimens may influence the kinetics of differentiation, DS remains an important toxicity across studies [118]. QTc prolongation represents a more agent-specific adverse effect, most prominently associated with revumenib, where largely low-grade events have been reported and are managed with electrocardiographic monitoring, electrolyte optimization, and avoidance of concomitant QT-prolonging medications [115]. In contrast, other menin inhibitors such as ziftomenib, bleximenib, and enzomenib have demonstrated minimal clinically significant QTc effects [118,120,121]. Hematologic toxicities, particularly neutropenia and thrombocytopenia, are most pronounced when menin inhibitors are combined with venetoclax-based regimens and typically require dose adjustments, growth factor support, and modified venetoclax schedules. Other nonhematologic adverse events, including gastrointestinal symptoms, fatigue, and mild transaminase elevations, are generally low-grade and rarely lead to treatment discontinuation. Overall, with appropriate monitoring and supportive care, the toxicity profile of menin inhibitors appears manageable and consistent with their mechanism of action.

## 7. Mechanisms of Resistance to Menin Inhibitors

**Figure 4:**
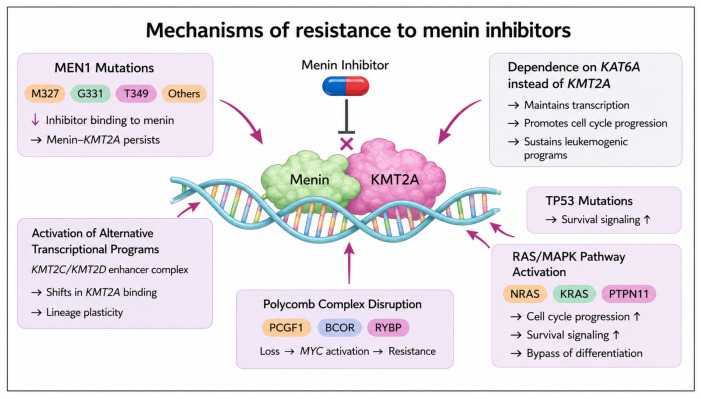
Overview of the molecular mechanisms of resistance to menin inhibitors.

Despite encouraging clinical activity, acquired resistance to menin inhibition has emerged as an important therapeutic challenge. Reported mechanisms include alterations affecting the menin protein itself as well as adaptive pathways that allow leukemic cells to bypass menin–KMT2A-dependent transcriptional programs (Figure 4).

### 7.1. On-Target Resistance

The primary on-target resistance mechanism is the acquisition of somatic *MEN1* mutations that create steric clashes impairing menin inhibitor binding while preserving the menin–KMT2A interaction [134,135]. This phenomenon has been most clearly documented in the setting of revumenib exposure, where *MEN1* mutations emerged in ~40% of patients receiving monotherapy, with recurrent hotspots at M327 (M327I/V), G331 (G331R/D), T349M, and S160 (S160C/T) [110,135]. Similar *MEN1* mutations were reproduced in xenograft models in response to different menin inhibitors, supporting their emergence through therapy-driven selection rather than stochastic background alteration [136]. With other menin inhibitors, resistance mutations have been less frequently reported. In the KOMET-001 trial, only one patient receiving ziftomenib developed a known resistance mutation (*MEN1* M327I) [118], while bleximenib, enzomenib, and other agents have not yet had *MEN1* resistance mutations reported in published trial data. However, this apparent difference should be interpreted with caution given the more recent introduction and less mature clinical data of these agents compared to revumenib. In vitro, ziftomenib retains biochemical activity against certain *MEN1* variants (G331R and T349M) but not against M327I or M327V. Mutations affecting M327 are particularly disruptive, impairing binding of multiple menin inhibitors, while other mutations may confer differential resistance across compounds [118,136]. Identification of the specific *MEN1* mutation may eventually help inform menin inhibitor selection as molecular profiling becomes more widely available and resistance patterns across inhibitors, doses, and combinations are better characterized.

### 7.2. Off-Target Resistance

While on-target resistance directly compromises inhibitor binding through mutations at the menin-drug interface, resistance to menin inhibition can also arise through off-target transcriptional and epigenetic adaptation rather than menin mutations, enabling leukemic cells to bypass *KMT2A*-dependent transcriptional programs despite preserved drug-target engagement [137,138]. In this setting, the inhibitor remains pharmacologically active at its target-site, but downstream leukemogenic expression is maintained through alternative mechanisms, rendering menin inhibition insufficient.

#### 7.2.1. KMT2A-Independent Resistance Through Transcriptional and Epigenetic Adaptation

One mechanism involves disruption of polycomb repressive complexes (PRC), particularly the non-canonical PRC1.1 complex [139,140]. PRC1.1, composed of proteins including BCOR, RING1B, KDM2B, and PCGF1, plays an important role in hematopoietic regulation and *HOXA9-*associated transcriptional programs [141]. Loss-of-function mutations in this complex have been associated with adverse prognosis in AML [142]. In this context, depletion of PRC1.1 components such as PCGF1 or BCOR can promote activation of alternative *HOXA9*/*MEIS1*-independent leukemogenic pathways, including *MYC*-dependent pathways [138,140].

Moreover, epigenetic alterations affecting components of the tumor-suppressive *KMT2C*/*KMT2D*–*UTX* complex can impair activation of differentiation associated transcriptional program following menin inhibition or chemotherapy, thereby attenuating therapeutic response and indicating that AML survival can be maintained independently of the canonical HOX axis [105].

The histone acetyltransferase KAT7, which cooperates with the menin–KMT2A complex to sustain oncogenic transcription, may also contribute to resistance when its activity persists despite menin inhibition [143,144].

#### 7.2.2. Clonal Evolution and Cooperating Mutations

Clonal evolution represents another important mechanism of acquired resistance to menin inhibition in *KMT2A*r and *NPM1*m leukemias. In heavily pretreated disease, leukemic cells may acquire cooperating mutations that drive proliferation independent of the original KMT2A-driven transcriptional program, resulting in attenuated sensitivity to menin inhibitors compared with earlier disease [144]. Consequently, leukemic persistence can occur despite on-target drug activity.

Genomic analyses reveal the emergence of cooperating mutations affecting *RAS* signaling and *TP53* pathways as well as chromatin regulators such as *KMT2C* and *KMT2D* which may enable subclones to proliferate outside of the menin–*KMT2A*-dependent programs [118,144]. Moreover, the intrinsic clonal heterogeneity of *KMT2A*r AML permits the selection and expansion of resistant subpopulations harboring mutations in the aforementioned signaling pathways [145]. Loss of *TP53* may further contribute to resistance by impairing p53–dependent apoptotic programs and promoting an apoptosis-resistant state characterized by dysregulation of BCL2 family proteins and increased dependence on MCL1 [146].

Collectively, these findings suggest that resistance to menin inhibition may arise through the expansion of genetically diverse subclones that bypass dependence on the menin–*KMT2A*–*HOXA* transcriptional axis, supporting the rationale for earlier use of menin inhibitors and rational combination strategies to evade clonal evolution [144].

### 7.3. Overcoming Resistance to Menin Inhibitors

Several strategies are being explored to overcome resistance to menin inhibition. A recent case report provided the first clinical evidence that switching from revumenib to bleximenib may have activity in a patient who developed an emergent *MEN1* M327I mutation after prolonged revumenib therapy [147]. However, larger studies are needed to determine whether menin inhibitor switching can reliably overcome on-target resistance. Combination therapy represents another promising approach to mitigating or circumventing resistance to menin inhibitors and is discussed in detail in Section 8.

## 8. Combination Strategies in Menin Inhibition: Mechanism and Application

Given the remarkable clinical activity of menin inhibitor monotherapy in R/R AML, considerable effort has focused on developing rational combination strategies to enhance efficacy and circumvent resistance. Several combination regimens are currently in clinical trials. Available efficacy and safety data are summarized in Table 2. These combinations can be broadly grouped based on the strength of the supporting evidence. The most clinically advanced strategies pair menin inhibitors with venetoclax, with or without hypomethylating agents (HMAs), or with intensive chemotherapy backbones. Ongoing trials such as SAVE, KOMET-007, and cAMeLot-2 highlight the efficacy of these combinations compared to other regimens. A second set of combinations that has less mature clinical data yet remains promising involves FLT3 inhibitor combinations. Multiple ongoing phase 1 trials are exploring FLT3 inhibitor combinations with menin inhibitors, with or without chemotherapy backbones. Other combinations such as CDK6 inhibitors [105] or agents that target epigenetic and transcriptional co-dependencies [148] are supported only by preclinical data so far.

Beyond enhancing antileukemic activity and accelerating response, combination strategies may also influence the biological consequences of menin inhibition, particularly the dynamics of leukemic differentiation. The addition of different combinations may modify this process by accelerating leukemic cytoreduction or enhancing apoptotic clearance of differentiating cells. Consistent with this hypothesis, emerging clinical data suggest that the incidence and severity of DS in several combination regimens may differ from that observed with menin inhibitor monotherapy, although cross-trial comparisons remain limited. At the same time, combination regimens introduce overlapping and additive toxicities including myelosuppression, hepatotoxicity, QTc prolongation, and infectious complications. These toxicities warrant careful evaluation as these strategies move into broader clinical use.

### 8.1. Combinations with Hypomethylating Agents and Venetoclax

The triplet combination of menin inhibitors, HMAs, and the oral BCL2 inhibitor venetoclax exhibits synergistic activity through complementary and largely non-overlapping mechanisms [162]. Menin inhibition reduces the expression of anti-apoptotic proteins such as BCL2 while promoting leukemic cell differentiation [162]. HMAs enhance apoptotic susceptibility by inducing the pro-apoptotic mediator NOXA, thereby priming leukemic cells for cell death [163]. Venetoclax, one of the most studied partners of menin inhibitors [164], then directly targets the residual BCL2 activity, further lowering the apoptotic threshold and facilitating mitochondrial apoptosis [165,166,167].

Accordingly, several trials are in progress evaluating the combinations of various menin inhibitors with venetoclax and HMA combinations, as summarized in Table 2 [149,152,156,159,161]. Early clinical experience suggests improved clinical and hematologic responses with manageable toxicity profiles. These findings support planned and ongoing phase 3 trials assessing the potential of triplet regimens of menin inhibitors, venetoclax, and HMAs to enhance therapeutic efficacy, mitigate resistance, and most importantly, improve clinical outcomes [168].

### 8.2. Combinations with Chemotherapy

Combining menin inhibitors with intensive induction chemotherapy is biologically attractive in *NPM1*m and *KMT2A*r AML, as cytotoxic chemotherapy provides rapid disease debulking while menin inhibition suppresses the leukemia-sustaining *HOXA*/*MEIS1* transcriptional program. The most mature frontline dataset comes from the phase 1a/b KOMET-007 study, in which ziftomenib combined with 7 + 3 induction demonstrated encouraging remission and MRD negativity rates, supporting advancement into the registrational phase 3 KOMET-017 trial [154]. Revumenib is also being evaluated with intensive induction and consolidation chemotherapy [153]. Additional menin inhibitors, including bleximenib, are planned for evaluation in combination with intensive chemotherapy [169]. Early clinical data on these combinations are summarized in Table 2.

### 8.3. Combinations with FLT3 Inhibitors

FLT3 inhibitors represent another class of agents being studied in combination with menin inhibitors. *FLT3* mutations frequently co-occur in *NPM1*m AML and at a lower frequency in *KMT2A*r AML [4,5,170]. In preclinical models, menin and FLT3 inhibitor combinations showed promising results [171,172], leading to more current clinical trials testing menin inhibitors with FLT3 inhibitors, presented in Table 3.

### 8.4. Combinations with IDH Inhibitors

*IDH1*/*2* mutations also frequently co-occur in *NPM1*m AML [170], and preclinical studies demonstrate that the combination of menin inhibitors with IDH inhibitors (ivosidenib or enasidenib) results in synergistic antileukemic activity [173]. Clinical data evaluating the combination remain limited to date.

## 9. Summary and Future Directions

Menin inhibitors have established menin dependence as a therapeutically actionable vulnerability in AML, particularly in leukemias driven by *KMT2A* rearrangements and *NPM1* mutations. Across clinical studies, these agents have demonstrated meaningful antileukemic activity with a generally manageable safety profile, supporting menin inhibition as an important emerging treatment strategy. Their clinical benefit in the R/R setting has been particularly notable, where responses may provide disease control or serve as a bridge to allogeneic stem cell transplantation.

Looking ahead, the next phase in the development of menin inhibitors will focus on their integration earlier in the disease course, most often as part of combination therapy. To date, combination regimens incorporating menin inhibitors in patients with ND AML have shown particularly encouraging clinical outcomes. In the R/R setting, the availability of menin inhibitors has already transformed the therapeutic landscape for many patients, frequently providing an effective bridge to subsequent therapies or alloSCT. As the field advances, MRD-guided strategies may further refine patient selection and facilitate more personalized treatment approaches.

Another important priority will be to better understand and overcome mechanisms of resistance to menin inhibitors, particularly those involving epigenetic alterations and clonal evolution. Although suppression of the *HOXA9*/*MEIS1* transcriptional program represents a central component of the mechanism of action of menin inhibitors, it does not fully account for the spectrum of clinical responses observed. Other genomic and transcriptional regulators are likely to influence menin-dependent leukemic programs and may contribute to both sensitivity and resistance. Identifying these factors could play an important role in treatment selection, sequencing, and monitoring. Ultimately, the future of menin-directed therapy will depend not only on improving the drugs themselves, but also on defining when to use them, in whom they are most effective, and how to best integrate them into a broader framework of biologically informed and patient-centered AML care.

## Figures and Tables

**Table 1:** Efficacy and Safety of menin inhibitor monotherapy in clinical trials.

Data	Menin Inhibitor	FDA Approval Status	Genetic Alteration	Overall Response Rate (%)	CR + CRh (%)	Grade ≥ 3 Differentiation Syndrome (%)	Grade ≥ 3 QT Prolongation (%)	CYP3A4 Interaction
More mature	Revumenib (SNDX-5613)	Approved	*KMT2A*r [115,116]	63	23	16	14	**+++**
		Approved	*NPM1*m [117]	47	23	23	13	
	Ziftomenib (KO-539)	Investigational	*KMT2A*r [118]	17	11	28	-	+
		Approved	*NPM1*m [119]	33	22	13	8	
Early reports	Bleximenib (JNJ-75276617)	Investigational	*KMT2A*r and *NPM1*m [120]	~45	~33	7	<1	Minimal so far
	Enzomenib (DSP-5336)	Investigational	*KMT2A*r [121]	59	23	NR	1	–
		Investigational	*NPM1*m [121]	54	23			

CR + CRh, complete remission + complete remission with partial hematologic recovery; FDA, Food and Drug Administration; NR, not reported. Symbols denote relative CYP3A4 interaction strength: +, limited interaction; +++, strong interaction. Icovamenib (BMF-219) and BN104 are currently under clinical investigation; available data remain preliminary and are insufficient for meaningful inclusion in this comparative table. Cross-trial comparisons should be interpreted with caution.

**Table 2:** Selected clinical trials of menin inhibitor-based combination regimens with intensive chemotherapy or venetoclax/hypomethylating agents.

Menin Inhibitor	Added Drug or Drug Combination	NCT Identifier	AML Subtype and Context	ORR (%)	Composite CR (%)	Differentiation Syndrome (%)	QTc Prolongation (%)
Revumenib	Azacitidine + Venetoclax [149]	BEAT AML NCT03013998 Phase 1	*KMT2A*r (ND AML)	100	89	19	44
			*NPM1*m (ND AML)	85	79		
	Fludarabine + Cytarabine [150]	AUGMENT-102 NCT05326516 Phase 1	*KMT2A*r, *NUP98*r, and *NPM1*m (R/R AML)	50	50	0	4% (≥G3)
	Venetoclax + (decitabine/cedazuridine) [151,152]	SAVE NCT05360160 Phase 1/2	*KMT2A*r, *NUP98*r, and *NPM1*m (R/R AML)	88	58	4 (≥G3)	8 (≥G3)
			*KMT2A*r and *NPM1*m (ND AML)	94	88	24	47
	Cytarabine + Daunorubicin [153]	ETCTN 10596 NCT05886049 Phase 1b	*KMT2A*r and high-risk *NPM1*m (ND AML)	88	88	0	0
Ziftomenib	Cytarabine + Daunorubicin [154]	KOMET-007 NCT05735184 Phase 1	*KMT2A*r (ND AML)	89	89	1 (≥G3)	3 (≥G3)
			*NPM1*m (ND AML)	94	84	0	0
	Venetoclax + Azacitidine [155,156]	KOMET-007 NCT05735184 Phase 1	*KMT2A*r (ND AML)				
			*NPM1*m (ND AML)	65	49	0 (≥G3)	3 (≥G3)
			*KMT2A*r (R/R AML)	33	22	0	0
			*NPM1*m (R/R AML)	65	49	2 (≥G3)	0
	FLAG-ida or LDAC [157]	KOMET-008 NCT06001788 Phase1	*KMT2A*r and *NPM1*m (R/R AML)				
Bleximenib	Cytarabine + Daunorubicin or idarubicin [158]	NCT05453903 Phase 1	*KMT2A*r and *NPM1*m (ND AML)	96	88	0	0
	Venetoclax + Azacitidine [159]		*KMT2A*r and *NPM1*m (ND AML)	92	85	4	0
			*KMT2A*r and *NPM1*m (R/R AML)	79 (at RP2D)	54 (at RP2D)		
	Venetoclax [160]		*KMT2A*r and *NPM1*m (R/R AML)	69	39	0	0 (≥3)
Enzomenib	Venetoclax + Azacitidine [161]	NCT04988555 Phase 1	*KMT2A*r and *NPM1*m (R/R AML)	83	56	6 (G2)	0

AML, acute myeloid leukemia; CR, complete remission; G2, grade 2; G3, grade 3; ND, newly diagnosed; ORR, overall response rate; RP2D, recommended phase 2 dose; R/R, relapsed or refractory. Gray-shaded rows denote ongoing clinical trials with no reported results to date.

**Table 3:** Current clinical trials of menin inhibitor-based combination regimens with FLT3 inhibitors.

Menin Inhibitor	NCT Identifier	AML Subtype and Context	FLT3 Inhibitor	Backbone
Revumenib	NCT06222580 Phase 1	*KMT2A*r or *NPM1*m + *FLT3-*ITD or TKD (R/R AML)	Gilteritinib	
	NCT06313437 Phase 1	*NPM1*m + *FLT3-*ITD or TKD (ND AML)	Midostaurin	Cytarabine + Daunorubicin
Ziftomenib	NCT06001788 Phase 1	*NPM1*m + *FLT3-*ITD or TKD (R/R AML)	Gilteritinib	
	NCT06769490 Phase 1	*NPM1*m*, KMT2A*r*,* and *NUP98*r (R/R AML)	Quizartinib	
	NCT05735184 Phase 1/2	*NPM1*m + *FLT3-*ITD (ND AML)	Quizartinib	Cytarabine + Daunorubicin

AML, acute myeloid leukemia; ND, newly diagnosed; R/R, relapsed or refractory. Gray-shaded rows denote that no chemotherapy backbone was used in those clinical trials.

## Data Availability

No new data were created or analyzed in this study. Data sharing is not applicable to this article.
